# Separation and degradation detection of nanogram-per-litre concentrations of radiolabelled steroid hormones using combined liquid chromatography and flow scintillation analysis

**DOI:** 10.1038/s41598-020-63697-y

**Published:** 2020-04-27

**Authors:** Roman Lyubimenko, Bryce S. Richards, Andrey Turshatov, Andrea I. Schäfer

**Affiliations:** 1 Institute of Microstructure Technology (IMT), Karlsruhe Institute of Technology (KIT), Hermann-von-Helmholtz-Platz 1, 76344 Eggenstein-Leopoldshafen, Germany; 2 Institute for Advanced Membrane Technology (IAMT), Karlsruhe Institute of Technology (KIT), Hermann-von-Helmholtz-Platz 1, 76344 Eggenstein-Leopoldshafen, Germany; 3 Light Technology Institute (LTI), Karlsruhe Institute of Technology (KIT), Engesserstrasse 13, 76131 Karlsruhe, Germany

**Keywords:** Chemical engineering, Environmental chemistry, Analytical chemistry

## Abstract

Detection of micropollutants such as steroid hormones occurring in the aquatic environment at concentrations between ng/L and µg/L remains a major challenge, in particular when treatment efficiency is to be evaluated. Steroid hormones are typically analysed using mass-spectrometry methods, requiring pre-concentration and/or derivatisation procedures to achieve required detection limits. Free of sample preparation steps, the use of radiolabelled contaminants with liquid scintillation counting is limited to single-compound systems and require a separation of hormone mixtures before detection. In this work, a method was developed coupling ultra-high-pressure liquid chromatography (UHPLC) with flow scintillation analysis (FSA) for separation and detection of radiolabelled estrone, 17ß-estradiol, testosterone and progesterone. Adjustment of the flow rate of scintillation liquid and UHPLC mobile phase, gradient time, column temperature, and injection volume allowed the separation of steroid hormones and degradation products. The limit-of-detection (LOD = 1.5–2.4 ng/L) and limit-of-quantification (LOQ = 3.4–4.3 ng/L) for steroid hormones were comparable with the current state-of-the-art technique (LC-MS/MS) for non-derivatised compounds. Although the method cannot be applied to real water samples (unless spiked with radiotracers), it serves as a useful tool for the development of water treatment technologies at laboratory scale as demonstrated via: i) adsorption on polymer-based spherical activated carbon, ii) retention in nanofiltration, iii) photodegradation using a photocatalytic membrane.

## Introduction

Water-borne trace contaminants such as pharmaceuticals, personal care products, and pesticides – collectively known as micropollutants – remain a serious issue due to the persistence and toxicity of such compounds^1^. The endocrine-disrupting effect of some micropollutants can cause drastic changes in the central nervous system as well as gender differentiation problems in wildlife and humans^2,3^. Steroid hormones, in particular naturally-occurring estrone (E1) and 17ß-estradiol (E2), exhibit the most potent endocrine-disrupting effect^4^. Other important steroid hormone intermediates are testosterone (T) and progesterone (P), whose relative estrogenic activity is two orders of magnitude lower than that of E2^5^. Indeed, E2 was demonstrated to produce an endocrine-disrupting effect even at sub-nanogram-per-litre concentrations^6^.

Given the pollutant type and technology employed, the removal efficiency of conventional wastewater treatment plants may vary greatly^7–10^. The incomplete removal of steroid compounds may result in a significant environmental impact caused by residual concentrations and synergistic effect of complex hormone mixtures^9,11^. Increased concentrations of steroid hormones (in particular E1 and E2) have been detected in surface waters (up to 137 ng/L of E2)^12,13^ and groundwaters (up to 147 ng/L of E2)^14^ as well as in the effluent of wastewater treatment plants (up to 143 ng/L of E1)^10,15^. The origins of the steroid hormones are mostly anthropogenic, with the most prominent example being their excretion due to hormonal therapies or the use of contraceptive pills^16^. With legislative control of micropollutants being still under development, a recent European Parliament Decision (EU) 2018/840 includes certain steroid hormones in the watch list of substances for close monitoring, including 17α-ethinylestradiol, E1, and E2^17^.

To meet stringent quality standards (maximum concentration of 0.4 ng/L for E2)^18^, advanced water treatment technologies need to be used^19,20^. However, the removal of water-borne micropollutants is closely intertwined with the sensitivity of detection methods^21^. The detection and quantitation of micropollutants with conventional mass-spectroscopy methods involves additional sample preparation steps, such as derivatisation and pre-concentration, to achieve better sensitivity. Due to the non-polar nature of steroid hormones, the ionisation of non-derivatised, low-concentration estrogens in conventional electrospray ionisation mode is rather low^22^. A derivatisation step, in turn, transforms analytes to compounds with an additional moiety to improve ionisation^23^ and, if needed, volatility and better thermal stability^24^. As increasing the injected analyte mass on column is desired, pre-concentration (enrichment process) may be performed using extraction techniques, such as solid-phase extraction (SPE). These require often the large sample volumes that may not be available in laboratory experiments. Further, the above procedures not only extend the analysis time and reduce the sample throughput^25^, but may also become an additional source of error due to variation of recovery^26^ or incomplete derivatisation^27^.

The two most powerful analytic techniques for steroid hormones are either separation-based gas chromatography (GC) or liquid chromatography (LC), both coupled with tandem mass spectrometry (MS/MS)^28,29^. The GC-MS/MS technique exhibits excellent chromatographic resolution^25^ and extremely low limits-of-detection (LOD) of estrogens, down to 0.1 ng/L after pre-concentration and derivatisation^12^. Despite these advantages, the GC-MS/MS technique remains less attractive for water analysis, due to the requirement of off-line sample preparation procedures like pre-concentration and derivatisation.

Instead, LC-MS/MS has become the preferred analytical method for hormone compounds with a wide range of polarity^26,30^, given the short analysis time and high throughput of samples not requiring derivatisation step^24,25^. After additional pre-concentration with SPE and, if desired, derivatisation of analytes, the limit-of-quantification (LOQ) using LC-MS/MS may be as low as 0.17 ng/L for E2^22^. Usually, such low reported values can be achieved through off-line SPE procedures and require a large sample volume (0.25–4 L) of the surface water^31–33^. On-line SPE methods developed in the last decade allow the loading of low volume samples (<1–10 mL) at LOQs as low as 0.5 ng/L^34–36^. However, the challenges and discrepancies in compound recovery and the errors introduced by the SPE cartridge remain^37,38^. Without prior extraction or derivatisation of steroid hormones, the sensitivity of state-of-the-art LC-MS/MS instruments is estimated to be at least 30 times higher (LOQ of 5 ng/L for E2)^39^. Nevertheless, the analysis errors at low concentrations (1–10 ng/L) achieved in case of high removal (90–99%) may jeopardise the quality of data for the treatment of hormone-containing waters. Thus, laboratory studies that involve measurement of low-volume samples (<10 mL) with residual concentrations of micropollutants (1–10 ng/L) remain difficult.

The method of liquid scintillation counting (LSC) has been used extensively in life sciences for the determination of isotope-labelled steroids^40^. The principle of LSC is based on measuring the photon emission from the energy transfer of radionuclide decay to a scintillation liquid^41^. The long-lived, low-energy β-emitting ^3^H-(tritium) and ^14^C-(carbon-14) isotopes are the most frequently used radiolabels. Tritium-labelled compounds are attractive due to their highest specific activity, easier handling and simple synthesis^42^. Although scintillation methods cannot be applied to real (natural) water samples that do not contain radiotracers, these methods can serve as a useful analytical tool capable of detecting ng/L concentrations and requiring low sample volumes^43^. In terms of sensitivity, such a method offers one of the lowest LOD’s for micropollutants (0.1–0.9 ng/L for E1 and E2)^44,45^. Despite the high sensitivity and accuracy for low-activity samples^46^, the LSC method lacks the ability to separate mixtures of analytes or detect the degradation of a compound as the measured ^3^H-bearing entity.

A classical approach to solve the problem of the mixture analysis is to use HPLC separation with subsequent fractionation of eluate and off-line analysis of samples with LSC. Besides the fact that the manual handling of hundreds of samples greatly increases the analysis time^47^, the lack of volume of collected fractions worsens the peak resolution. The aforementioned problems (long analysis time and poor resolution of the reconstructed radiochromatograms) can be solved, by using, for example, off-line microplate scintillation counting (MSC) coupled with rapid fractionation (1–2 s/well)^48^. The benefits of the MSC method, however, diminish as the solvent evaporation stage may lead to a loss of volatile components^49^, hence requiring the determination of recovery of radioactive compounds^50^.

Flow scintillation analysis (FSA), free of any sample preparation or manual operations, measures the radioactive activity in-line in a flow-cell. As an in-line detector, it can be coupled with HPLC, rendering it well-suited for the analysis of radiolabelled compounds in mixtures or the monitoring of their degradation. FSA is one of the preferred techniques for metabolite and drug analysis in biological or medical sciences^42,49^, where a high throughput of relatively low-concentrated samples is required^47^. There are several reports of coupling FSA with HPLC separation. For example, HPLC connected with FSA and MS was used by Tykva *et al*. for the separation and detection of biodegradation products of juvenoid diastereoisomers (hormones)^51^. Under acetonitrile-water gradient elution, the 200 μL of degradation products were injected, monitored using the ultraviolet (UV) detector and quantified using an FSA-radiodetector. The LOD (defined as a signal-to-noise ratio of 3) was at a concentration of 2 μg/L (equivalent to 150–200 Bq). Abdel-Khalik *et al*. examined the metabolites of ^14^C-cholesterol) using an HPLC-FSA technique, with the method developed using unlabelled hormones and measured by a UV detector^52^. After the external SPE step with an enrichment factor of 5, the LOD for the resulting method (0.2 mL injection) was at 71.5 μg/L. Pettersson *et al*. used the HPLC-FSA method in order to detect the metabolites of 5α-androstane-3α,17β-diol^53^. After the 50-times enrichment (from 5 to 0.1 mL), the analytes of 3α-adiol (0.1 mL, 15.2 mg/L) were separated with a methanol/water gradient elution and analysed using an FSA-detector, although no LOD value was reported^53^.

Given the above studies using the HPLC-FSA method, the key challenge remains on how to extend the LOD from the μg/L range to ng/L of radiolabelled hormones. In the last two decades, the development of columns with small-size (less than 2 μm) particles brought an era of ultra-high performance liquid chromatography (UHPLC) known for high operating pressure, increased resolution and reduced runtime^54^. However, studies reporting the use of UHPLC-FSA analysis of radiolabelled hormones could have to date not been published. To explain the lack of research, it must be noted that low peak volumes (10–20 µL) achieved with state-of-the-art UHPLC columns are not well compatible with large-volume flow cells (150–1000 µL) of FSA-detectors^55^. Such combinations result in a severe peak dispersion that causes a loss of sensitivity and poor peak resolution. Yet, the method appears to offer potential to achieve very low detection limits, while achieving the separation of hormones in mixtures and the quantification of degradation products.

Thus, the outstanding research questions are as follows: (i) how can steroid hormone micropollutants at ng/L concentrations be detected in sample volumes of <1 mL?; (ii) how can the peaks of eluting hormones in mixtures be resolved using the UHPLC-FSA method?; and (iii) how can hormone mixtures and their degradation products be quantified?

In this work, the results of the separation of four steroid hormones (estrone, estradiol, testosterone, and progesterone) in ng/L concentrations are presented. The method development includes the variation of both liquid scintillation (LS) pump and UHPLC flow rates, gradient time, column temperature, the volume of injection, as well as the estimation of the *R*_*S*_ values, which are the key figure-of-merit in the analytical separation process. The sensitivity of the method (evaluated via LOQ) was compared with those reported for LC-MS/MS analysis of non-radiolabelled estrogens.

To demonstrate the applicability of this method in water treatment research, we conducted three examples of using the UHPLC-FSA method for the quantification of steroid hormone mixtures: (i) adsorption studies with hormone mixtures using polymer based spherical activated carbon (PBSAC)^56^; (ii) membrane filtration experiments using mixtures in nanofiltration^57^; and (iii) degradation products after photocatalysis.

## Methods

### Chemicals

Radiolabelled [2,4,6,7–^3^H(N)]-estrone (E1, 1 mCi, 3.69·10^12^ Bq/mmol, Batch No 2165951), [2,4,6,7-^3^H(N)]-estradiol (E2, 1 mCi, 3.48·10^12^ Bq/mmol, Batch No 2354825), [1,2,6,7-^3^H(N)]-testosterone (T, 1 mCi, 3.53·10^12^ Bq/mmol, Batch No 2151380) and [1,2,6,7-^3^H(N)]-progesterone (P, 1 mCi, 3.57·10^12^ Bq/mmol, Batch No 2136265) were supplied in ethanol (PerkinElmer LAS GmbH, Germany). The structure of the chemicals and their properties are summarised in Supplementary Table S1. Diluted tritium water (HTO, volume activity of 2.4· 10^10^ Bq/L) was obtained from the Institute for Technical Physics (ITEP), within KIT. UHPLC-grade methanol was purchased from Fischer Scientific (Germany). Ultrapure water (Milli-Q, type 1, Merck Millipore; >18.2 MΩ/cm at 25 °C) was used in all experiments. The feed solutions of steroid hormones demonstrated in application examples were prepared from the different E2 batch (3.26·10^12^ Bq/mmol, Batch No 2526124) in a background solution containing 1 mM of sodium bicarbonate (99.7%, Bernd Kraft, Germany) and 10 mM of sodium chloride (99.9%, VWR Chemicals, Germany).

### Sample preparation

A stock solution of each steroid hormone (10 µg/L) was prepared by dilution with ultrapure Milli-Q water of the as-supplied solution. The hormone concentration in this native solution (*c*_*nat*_) was calculated from Eq. (1)

1 $${C}_{nat}=\frac{{a}_{tot}\cdot M}{{a}_{s}.{V}_{nat}},$$

where *a*_*tot*_ is the total activity in purchased solution of hormone (Bq), (1 mCi = 3.7·10^7^ Bq), *a*_*s*_ is the specific activity of hormones in the purchased solution (Bq/mmol) (variability of activity in stock solution was not observed as long as the same batch of purchased solution was used), *M* is the molecular weight of specific hormone (g/mol) as reported in Supplementary Table S1, and *V*_*nat*_ is the volume of as-purchased hormone solution (L). The concentration of hormone (*c*_*H*_) is related to the volume activity (*a*_*V*_) via Eq. (2):

2 $${a}_{V}=\frac{{a}_{S}}{M}\cdot {C}_{H},$$

where *a*_*V*_ is the volume activity of a sample (Bq/L) and *c*_*H*_ is the mass concentration of hormones (g/L).

For each hormone, a series of solutions with concentrations of 1, 2, 5, 10, 20, 50, 100 ng/L were prepared via 10^3^-fold to 10-fold dilution of the stock solution with Milli-Q water. The resulting standards along with the blank samples (n = 12 injections) were analysed with the UHPLC-FSA method at conditions denoted as “standard” in Supplementary Table S3.

To construct the calibration curves (n = 5 points), the integrated radiochromatogram peak areas (*y*_*P*_, counts) of standard solutions were plotted against the concentration. All standards prepared for calibration curves were stored in the fridge at 4 °C before analysis.

### Analytical methods

**Figure 1:**
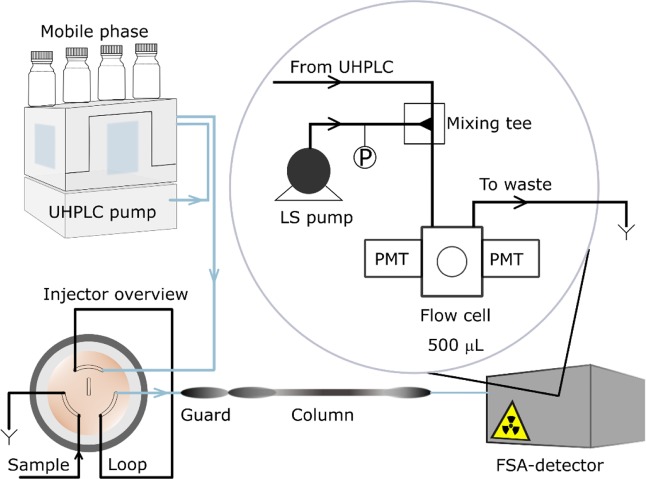
Schematic of the ultra-high-performance liquid chromatography-flow scintillation analysis (UHPLC-FSA) system with the internal configuration of the FSA-detector.

The samples were kept inside the autosampler (Flexar FX UHPLC, Perkin Elmer) at a temperature of 4 °C and then injected onto the column thermostated in an LC Column Oven (Flexar LC, Perkin Elmer). The guard-column (C18, SecurityGuard Ultra, Phenomenex) was installed for protection of UHPLC column packing. To increase the separation efficiency and, thus, maximise the peak resolution, a long column with a core-shell C18 stationary phase (1.7 µm, 100 Å, 150 ×2.1 mm, Kinetex, Phenomenex) was chosen. The conventional C18 bonded phase was chosen due to good resolution and retention of steroid compounds^58^. The methanol-water gradient elution was realised by the UHPLC pump (Flexar FX-20, Perkin Elmer). The choice of mobile phase was made from the preliminary tests showing a better resolution of E2 and T peaks than that with acetonitrile-water elution. The time of changing of mobile phase composition (gradient time) was adjusted for the improvement of the peak resolution during the method development. Prior to the liquid flow cell of the radiodetector, the UHPLC eluate was mixed in a static mixing tee with the liquid scintillation (LS) cocktail (Ultima Flo M, PerkinElmer LAS GmbH, Germany). The LS cocktail was delivered by the internal volume scintillator pump of flow scintillation analyser (FSA, Radiomatic 625TR, 500-μL liquid flow cell) (Fig. 1). The activity of the resulting mixture was monitored as a count rate using an FSA-detector. The working principle is based on the photon counting by means of two photomultiplier tubes (PMT) and a coincidence counting circuit^41^.

Based on the calibration curves obtained, *y*_*P*_ (counts/min) can be converted to the concentration *c*_*H*_ and volume activity *a*_*V*_ via Eq. (3) and Eq﻿. (4):

3 $${C}_{H}=\frac{{y}_{P}-{y}_{o}}{S},$$

4 $${a}_{V}=\frac{{a}_{S}}{M\cdot S}\cdot ({y}_{P}-{y}_{0}),$$

where *S* is the slope of the calibration curve of hormones (−) and *y*_0_ is the intercept of the calibration curve (counts/min).

The main differences of a FSA-detector are that both the sensitivity and the resolution of the peaks for the former rely on the size of a flow cell, the ratio between LS cocktail and HPLC eluate, and the update (integration) time of detector^59^. Here, a flow cell volume of 500 μL was chosen as a compromise between the achievable accuracy of measurement due to the higher volume and the dispersion of eluting peak inside the flow cell. Additionally, the splitter and diverter lines inside the FSA-detector were bypassed via connecting the column outlet directly to the flow-cell.

The effect of LS flow rate on the chromatographic parameters (retention time, shape and area of a peak) was carried out with the E2 samples of 100 ng/L and is presented in Supplementary Fig. S1. The narrowest peaks, estimated using the full-width-at-half-maximum (FWHM) values, were observed at LS flow rates greater than 4 mL/min. This trend could be explained by the reduced peak dispersion inside the flow cell at high flow rates due to a decrease of residence time^55^. Thus, to achieve the highest peak resolution, an LS flow rate of 4 mL/min was fixed for all remaining experiments.

The radiochromatograms were obtained from the ProFSA v.3.4.3 software (Perkin Elmer), with a counting time of 6 s to ensure an accurate measurement. The peak analysis (retention time and width of peaks) was performed in OriginPro 2017 software (OriginLab).

### Error estimation

The error bar of count rate ($\triangle {\rm{CPM}}$) in quantification example studies was estimated via error propagation method considering the uncertainties of sample preparation (∆Prep = 5%), the experimental system (∆S = 1, 8 and 9%) for adsorption, filtration and photocatalysis studies, UHPLC system (∆UHPLC = 1%), and FSA-detector (∆Det =12–16%). More details on error estimation can be found in Supplementary Information.

### Estimation of chromatography resolution and sensitivity

The degree of chromatographic separation achieved was assessed using the resolution *R*_*S*_ value and capacity factor (*k*), which were calculated in accordance with Eq. (5) and Eq. (6)^60^:

5 $${R}_{S}=1.18\frac{{t}_{r2}-{t}_{r1}}{FWH{M}_{1}+FWH{M}_{2}},$$

6 $$k=\frac{{t}_{r}-{t}_{0}}{{t}_{0}},$$

where *t*_*r1,2*_ are the retention times of analytes of interest (min) and *FWHM*_*1,2*_ are the full-width-at-half-maximum values of the analyte peaks (min). A minimum required *R*_*S*_ value for adjacent peaks was recommended to be *R*_*S*_ > 2, taken from U. S. Food and Drug Administration (FDA) guideline^61^, *t*_0_ is the dead time of the system, which will be determined later (section “Use of tritiated water in UHPLC-FSA analysis”).

Estimation of system response (expressed as peak area in counts/min) at the limit-of-detection (*y*_*LOD*_) and the limit-of-quantification (*y*_*LOQ*_) was conducted using the Eq. (7) and Eq. (8)^62^:

7 $${y}_{LOD}={y}_{B}+3\cdot {\sigma }_{B},$$

8 $${y}_{LOQ}={y}_{B}+10\cdot {\sigma }_{B},$$

where *y*_*B*_ is the mean value of blank signal (counts/min) and *σ*_*B*_ is the standard deviation of the blank signal (counts/min).

## Results

### Separation of hormone mixtures

**Figure 2:**
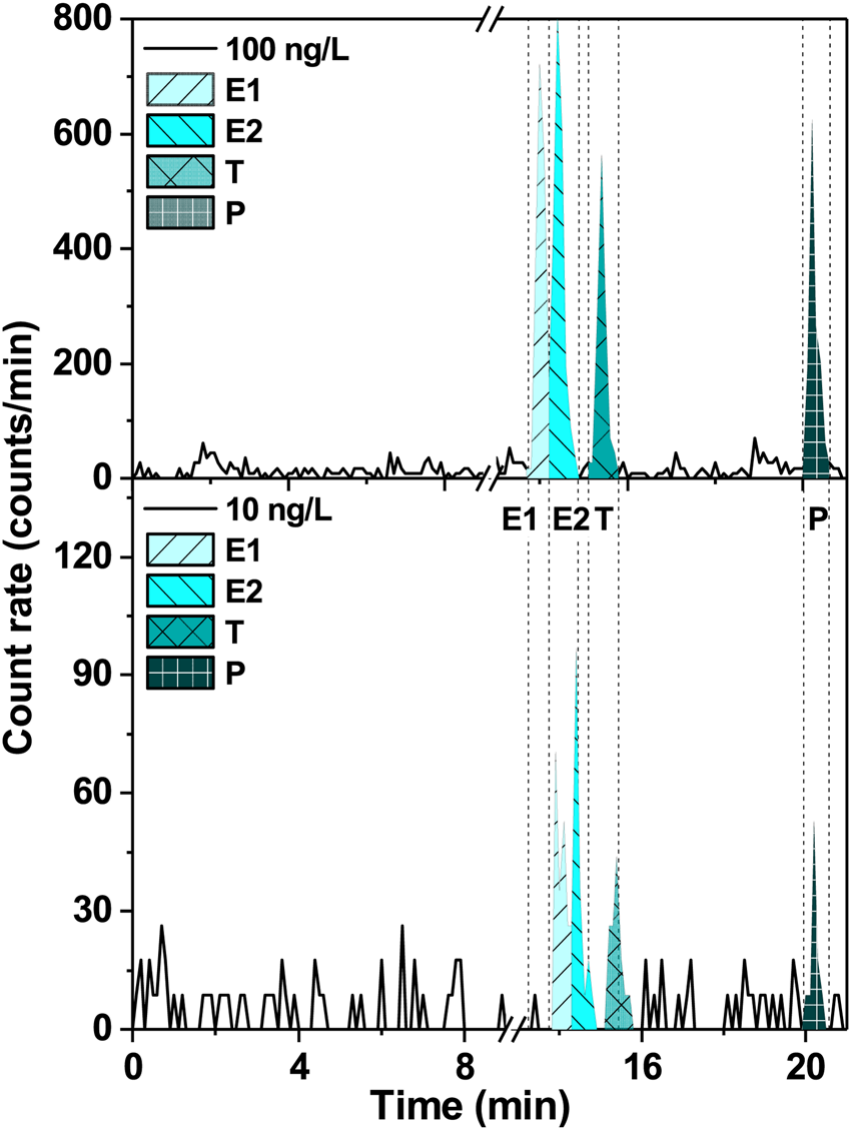
UHPLC-FSA radiochromatograms of a mixture of ^3^H-labelled E1, E2, T and P hormones at two different concentrations (10 and 100 ng/L of each hormone). The elution conditions are presented in Supplementary Table S3 as “standard conditions”.

The results of separation of hormones at ng/L concentrations were obtained by injecting the mixtures of E1, E2, T, and P, at 10 and 100 ng/L of each hormone for analysis with the UHPLC-FSA method. Figure 2 demonstrates that all the four peaks of E1, E2, T, and P – both at concentrations of 10 and 100 ng/L – were visible despite the strong peak dispersion. The calculated resolution of adjacent (close-eluting) peaks, namely R_S_ (E1 - E2) = 1.1, R_S_ (E2 - T) = 2.6 and R_S_ (T - P) = 15.3 confirmed the baseline separation for E1 or E2, T and P based on the recommendation *R*_*S*_ > 2^61^. Due to the similar interaction of E1 and E2 with the C18 stationary phase, the current method resolution was not sufficient to completely avoid their co-elution (*R*_*S*_ < 2). The key results of UHPLC-FSA separation of hormones are summarised in Table 1. High values of capacity factor (*k*) demonstrated that all hormones are highly retained exceeding the minimum required *k *> 2^61^.

**Table 1:** The UHPLC-FSA separation results of hormone mixture (each 100 ng/L).

	Time (min)	Capacity factor (−)	FWHM (min)	Area (counts/min)
E1	14.0	6.8	0.21	200 ± 18
E2	14.4	7.0	0.24	211 ± 18
T	15.4	7.6	0.21	122 ± 13
P	20.2	10.2	0.16	128 ± 13

### Use of tritiated water in UHPLC-FSA analysis

The separation results of radiolabelled steroid hormones were achieved using the method with a pre-selected column type, eluent and elution type. However, further optimisation of the UHPLC-FSA method conditions was required. The extra-column volume affects the dispersion and hence the resolution of the eluting peaks^58^, thus it was essential to estimate and reduce the system dead volume (*V*_0_, the volume between the injection point and FSA-detector) prior to optimisation of separation parameters. One approach for determining *V*_0_ involves the injection of unretained compounds. When dealing with radiochemicals, diluted tritiated water (HTO) is ideally suited for that purpose. Thus, the behaviour of HTO samples exhibiting different activity was investigated, with radiochromatograms of such tracer compounds illustrated in Supplementary Fig. S2.

HTO elutes at retention time *t*_*r*_ = 1.8 ± 0.1 min, with the peak area increasing proportionally to the activity. The retention time of HTO was used as the dead time of the system (*t*_0_, the transition time of dead volume) for calculation of the capacity factor *k* presented in Table 1. From the *t*_0_-value, the dead volume of the system was calculated to be 0.45 mL (of that, 0.26 mL is the estimated column volume). Compared to the conventional UHPLC values (μL range)^58^, the calculated extra-column volume (∼0.2 mL) was found to be excessive. It thus may result in post-column dispersion from the mixing parts and flow-cell of FSA-detector and ultimately the absence of peak resolution. The work-around of this situation could be an improvement of the chromatographic resolution by changing UHPLC operation parameters.

### Optimisation of chromatographic conditions for the steroid hormone separation

**Figure 3:**
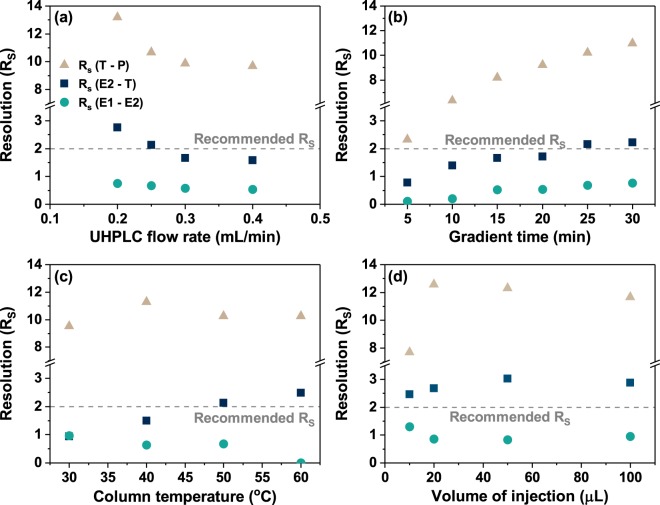
Influence of (**a**) UHPLC flow rate, (**b**) methanol-water gradient time, (**c**) column temperature, (**d**) volume of injection on the peak resolution of E1–E2, E2–T and T–P pairs obtained from a single hormone elution. A recommended *R*_*S*_ > 2 for adjacent peaks was marked with a dashed line.

The method development was based on a systematic investigation of the effect of varying four parameters, namely UHPLC flow rate, gradient time, column temperature, and volume of injection, which are presented in detail in Supplementary Table S3. Such an approach resulted in a solid understanding of how the separation of four hormones – E1, E2, T, P – are affected by i) flow rate; ii) gradient time; iii) column temperature; and iv) volume of injection. The radiochromatograms of the achieved results are demonstrated in Supplementary Figs. S3–S6. Any changes in retention time and peak shape of analytes were reflected in the *R*_*S*_ value. From the analysis of obtained radiochromatograms for single hormones, the resolution values of adjacent (neighbouring) peaks, *R*_*S*_ (E1 - E2), *R*_*S*_ (E2 - T) and *R*_*S*_ (T - P), were estimated (see Fig. 3a–d).

Figure 3a demonstrates the effect of the UHPLC flow rate on the peak resolution, which increased for all hormone pairs as the eluent flow rate decreased. On the basis of the *R*_*S*_ > 2 goal, it is observed that the successful resolution of adjacent peaks of T and P, E2 and T at flow rates less than 0.25 mL/min could be achieved. It was not possible to completely resolve the peaks of E1 and E2 due to their partial co-elution. Hence, a flow rate of 0.25 mL/min was chosen for the separation of hormones to address the trade-off between the analysis time and the peak resolution of E2 and T.

In contrast to the eluent flow rate, the gradient time affects the capacity factor by shifting only the retention time of analytes of interest. The gradient profiles with the changes of methanol content in the mobile phase over the LC runtime can be found in Supplementary Fig. S4a. The correlation between the observed changes in radiochromatograms with the *R*_*S*_ values of neighbouring peaks is performed in Fig. 3b. The *R*_*S*_ values for all tested hormone pairs increased as the methanol-water gradient time increased. At gradient times >25 min, the analyte pairs of E2 and T were shown to be potentially resolved (*R*_*S*_ > 2), while the peak resolution of T – P pair was high even at the shortest gradient time. The close elution of E1 and E2 (*R*_*S*_ < 2) was not significantly affected by the increase of gradient time. As a trade-off between the analysis time and resolution of E2 and T pair, the gradient time of 25 min was chosen for the separation of steroid hormones.

The oven-controlled temperature of the column may affect the viscosity of the solvent, diffusion of analytes and selectivity of the column. Hence, the influence of column temperature on the column selectivity (expressed via analyte retention times) and peak shape was investigated for tested hormones (see Supplementary Fig. S5 and Fig. 3c). In general, *R*_*S*_ values did not significantly change with increasing column temperature. The peaks of the T - P pair were resolved at all tested temperatures, while the E1 - E2 pair was not possible to resolve. However, a major gain in the peak resolution for the E2 - T pair was observed at higher temperatures, which defined the choice of the column temperature at 50 °C due to the successful separation of E2 - T analyte pair.

As the injection volume is proportional to the mass of analyte reaching the detector and affecting the LOD of the method, a series of injection volumes were tested. As demonstrated in the radiochromatograms in Supplementary Fig. S6, the injection volume does not change the analyte retention. In turn, large injection volumes leading to extra-column band broadening (observed as an increase of peak width) are known to affect the peak resolution. However, the different volumes of injection did not demonstrate the change of the peak resolution expressed as *R*_*S*_ value (Fig. 3d). Hence, the volume of injection of 100 μL was chosen to achieve the highest sensitivity (by means of increasing the mass of analyte on column) without compromising the resolution of adjacent peaks.

### Estimation of LOD/LOQ

**Figure 4:**
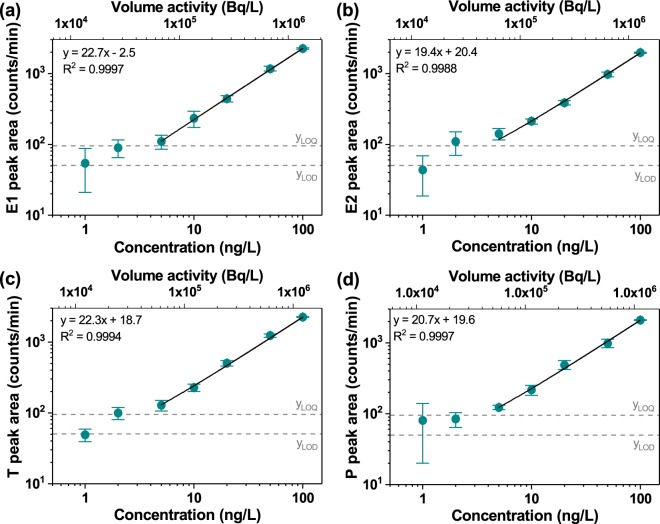
Calibration curves of (**a**) E1, (**b**) E2, (**c**) T and (**d**) P. The error bar was calculated as maximum error based on at least four repeats. The fitting (solid line) was performed in linear coordinates. The estimated peak areas at the limit of detection (*y*_*LOD*_) and the limit of quantification (*y*_*LOQ*_) were demonstrated as dashed lines. The details on the estimation of activity at LOD are given in Supplementary Information.

After the optimisation of separation parameters, the radiochromatograms of injected standards were obtained and presented in Supplementary Fig. S7. Based on peak areas obtained from these radiochromatograms, the calibration curves for each steroid hormone were constructed (Fig. 4).

The calibration curves for all four hormones demonstrated a good fit (R > 0.996) of linear regression in the concentration range of 5–100 ng/L. Based on blank sample injections, the FSA-detector response at LOD (*y*_*LOD*_) was calculated to be 53 counts/min. The intercept of calibration curves with *y*_*LOD*_ on presented graphs resulted in a LOD for tested hormones in the range of 2–4 ng/L. The LOD and LOQ values calculated for steroid hormones were summarised in Table 2. The LOQ values for steroid hormones presented in this study were comparable to or better than those obtained for non-derivatised estrogens using SPE-UHPLC-MS/MS (5–44 ng/L)^37,39,63^. Thus, the developed method at low sample volumes (<1 mL) appears to be competitive in terms of sensitivity with a LC-MS/MS technique. More importantly, it enables the use of LSC methods for testing of mixtures or degradation products.

**Table 2:** Parameters obtained from calibration curves of tested hormones.

	*y*_*Β*_ (counts/ min)	*σ*_*Β*_ (counts/ min)	*y*_*LOD*_ (counts/ min)	*y*_*LOQ*_ (counts/ min)	*S* (slope)	LOD (ng/L)	LOQ (ng/L)
E1	35	6	53	95	22.7	2.4	4.3
E2					19.4	1.7	3.8
T					22.3	1.5	3.4
P					20.7	1.6	3.6

### Quantitation of steroid hormones in different water treatment technologies

One of the main objectives was to develop a method able to overcome limitations of the LSC method. Those are i) the individual analysis of steroid hormones in mixture and ii) the separation and quantification of degradation products. The scope of potential applications for the developed method was demonstrated in three water treatment technology examples given below and is, of course, expandable to a wealth of research where micropollutant partitioning, removal and degradation is to be examined.

### Example 1. Adsorption studies

**Figure 5:**
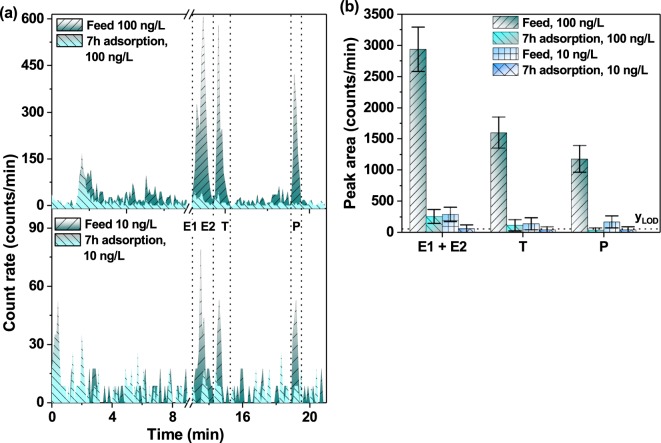
Adsorption experiments with the mixtures of E1, E2, T, and P (100 and 10 ng/L each). (**a**) UHPLC-FSA radiochromatograms, (**b**) the change of obtained peak areas for analytes after 7 h of adsorption experiment. The error bar was estimated via error propagation approach as presented in Supplementary Information.

As a first goal, the quantification of removal of multiple hormones prepared in one solution was demonstrated in adsorption studies. For that purpose, the samples collected after static adsorption experiments were obtained after 7 h shaking of a mixture of E1, E2, T, and P (each at 100 ng/L) with polymer-based spherical activated carbon (PBSAC)^64,65^. Then, the mixtures of hormones before and after 7 h adsorption experiments were compared (see Fig. 5). Details on experiment protocol and material characterization can be found elsewhere^56^.

It was shown using the LSC method that PBSAC achieves a high removal (more than 90%) of individual hormones such as E1, E2, T, and P (each 100 ng/L)^56^. Figure 5a demonstrates the successful resolution of the adjacent peaks of E2, T and P injected as a mixture, despite the partial co-elution of E1 and E2. The radiochromatograms also exhibited similar retention times both in the feed (100 and 10 ng/L of each hormone) and after the adsorption experiments. The resolution of eluting hormone peaks was not changed at lower concentrations. The advantage of this method is that any impurities or oxidation by-products (eluted at *t*_*r*_ = 1.5–3 min) were not retained by the column so that these would not be considered in the calculation of hormone removal. The hormone mixtures before and after adsorption experiments were separated and analysed in terms of removal based on the change of peak areas (as seen in Fig. 5b). Apart from partly co-eluting E1 and E2 and, thus, analysed together, the peak areas for each individual hormone before the experiment were compared with the ones after 7 h of adsorption with PBSAC. The removal of each hormone demonstrated the similar to the previously reported by Tagliavini *et al*. values of around 90%^56^, with further work showing a significantly higher removal.

### Example 2. Nanofiltration of hormone mixtures

**Figure 6:**
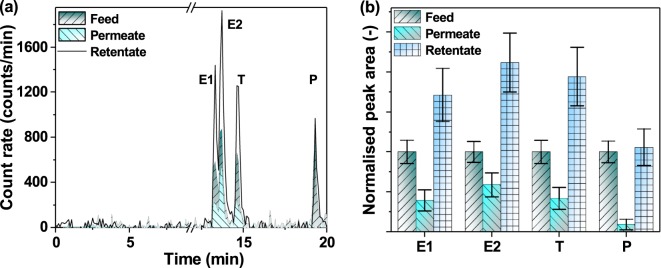
Nanofiltration membrane experiments with the mixtures of E1, E2, T, and P (100 ng/L each). (**a**) UHPLC-FSA radiochromatograms, (**b**) the normalised peak area for each analyte in feed, permeate and retentate samples. The permeate was collected after filtration of 700 mL volume. The error bar was estimated via error propagation approach as presented in Supplementary Information.

Nanofiltration studies were conducted in the dead-end stirred cell system^43,57,66^. The polyamide thin-film-composite nanofiltration membrane (NF 90, internal diameter of 7 cm) was used for filtering the mixture of E1, E2, T, and P. The further details on the filtration protocol and the system hydrodynamic conditions are given elsewhere^64,67^. The results of the analysis of collected feed and permeate samples are presented in Fig. 6a. The observed peaks were quantified that demonstrated the reduction of peak area for E1 (61%), E2 (41%), T (58%) and P (91%) in permeate samples after filtration of 700 mL of feed solution (Fig. 6b). In contrast, the retentate samples demonstrated an increase in peak area for E1 (71%), E2 (110%), T (94%) and P (6%) due to their rejection by the membrane. It should be noted that the NF90 membrane was reported to provide 70–80% removal of E2 solutions^64^. Understanding such differences between mixtures and single solutions require further studies.

### Example 3. Photocatalytic degradation of hormones

**Figure 7:**
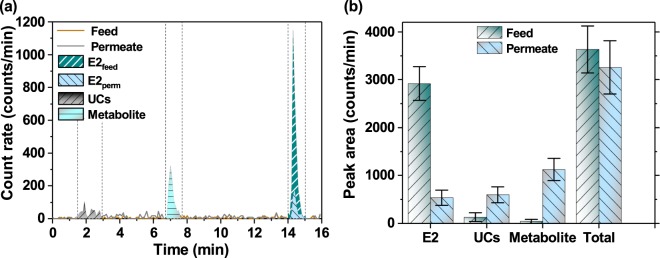
(**a**) The UHPLC-FSA radiochromatograms of feed and permeate solutions of E2 at 100 ng/L. (**b**) The change of peak areas for E2, unretained compounds (UCs), metabolite (new product) and total integrated area (*t*_*r*_ = 0–16 min). The error bar was estimated via the error propagation approach as presented in Supplementary Information.

In the photocatalytic degradation tests, the E2 hormone solution was continuously pumped through the poly(vinylidene-fluoride) (PVDF) membrane with immobilised on its surface Pd(II) meso-Tetra(pentafluorophenyl)porphine (PdTFPP)^68^. Upon simultaneous exposure to the warm-white light-emitting diode (SOLIS-3C, Thorlabs), the photocatalytic membrane produced species oxidising E2 molecules. The details on the preparation of the photocatalytic membrane and experimental protocol were reported in detail elsewhere^68^. To have the separation of photodegradation products shown, the permeate samples collected after photocatalytic degradation of E2 were analysed (Fig. 7a).

After the photocatalytic degradation, the changes in peak height of E2 hormone-containing permeate samples were observed. The peak area of E2 significantly reduced after the filtration through the light-exposed PdTFPP-PVDF membrane. A new peak of unknown metabolite eluting at *t*_*r*_ = 7 min was found in the permeate samples. Furthermore, multiple peaks of unretained compounds eluting close to the retention time of HTO (*t*_*r*_ = 1–3 min) were observed in the radiochromatogram of permeate samples.

As the quantfication of the separated hormones and their by-products in photodegradation experiments was pursued, the integrated areas under peaks before and after the experiment were compared (Fig. 7b). Via comparison of peak areas in feed and permeate samples, the removal of E2 (82%) and its conversion to unretained compounds (19%) and new product (39%) may be calculated. Despite the complicated mass balance for tritium-labelled compounds^42^, the total integrated areas representing the activity of samples in the feed and permeate were found to be in good agreement.

## Discussion

Although analytical techniques for the separation and detection of steroid hormone micropollutants exist, there is a lack of methods not requiring complex, multistage sample preparation and low sample volume to detect ng/L concentrations. The previous use of highly-sensitive scintillation methods, namely LSC analysis, lacked the ability of mixture and/or degradation product separation.

In this study, the UHPLC-FSA method was shown to offer the measurement of ^3^H-labelled compounds in small volumes without the prior pre-concentration at ng/L concentrations. The investigation of separation parameters showed that the flow rate and the gradient time had a strong influence on analyte retention. However, a limited effect on the peak resolution of E1 and E2 analytes was observed in view of their partial co-elution due to a similar interaction with the C18 column. Interestingly, the column temperature improved the selectivity only for the E2 - T pair, while no visible change was found for E1 - E2 and T - P pairs. Due to the low concentration of samples, the steroid hormone molecules were adsorbed at the head of a column, with the following desorption during the gradient elution. Thus, the volume of injection had no significant effect on the shape of peaks.

The LOD (1.5–2.4 ng/L) and LOQ (3.4–4.3 ng/L) of the developed method demonstrate the sensitivity for steroid hormones comparable with the modern LC-MS/MS and low sample volumes (5–44 ng/L)^37,39,63^. As a possible limitation, the size of a flow cell and counting time are likely to be a bottleneck of the FSA-method in terms of further LOD reduction^59^. Depending on the method objective, either the sensitivity or the peak resolution (as trade-off parameters) can be further enhanced by variation of the flow cell geometry and residence time.

Three examples for applying the UHPLC-FSA method in water treatment technologies with sub-mL sample injection volumes were demonstrated. An inherent limitation of off-line scintillation methods in view of mixture analysis can be overcome with the UHPLC-FSA. A successful quantification of removal and retention of hormone mixtures in adsorption and filtration studies was demonstrated that may give insights into the mechanism of removal process in hormone mixtures. Radiochromatograms of collected samples after photocatalytic degradation studies showed both the qualitative and quantitative changes of radioactive compounds. Namely, the transformation of E2 into a new, more polar, hydroxylated product (E2 + ^1^O_2_ ⇒ E2–OH) was detected. Its structure is expected to be similar to that of compounds reported in the study of ^1^O_2_-mediated degradation of E2 with Rose Bengal as photosensitiser^69^. It was assumed that E2 partially converts to unretained compounds and the new product during photodegradation.

## Conclusions

A UHPLC-FSA method was developed to analyse the nanogram-per-litre concentrations of radiolabelled steroid hormones (LOD = 1.5–2.4 ng/L and LOQ = 3.4–4.3 ng/L). On the basis of the variation of HPLC and LS flow rate, gradient time, column temperature and volume of injection, the method was optimised in terms of peak resolution of steroid hormones. Their peaks were clearly visible on the resulted chromatogram, with *R*_*S*_ (E2 - T) = 2.6, *R*_*S*_ (T - P) = 15.3, and *R*_*S*_ (E1 - E2) = 1.1 observed as a partial co-elution.

The method was successfully applied in adsorption and filtration experiments for the quantification of hormone mixtures by means of comparison of integrated areas. At *t*_*r*_ = 7 min a new metabolite after photodegradation studies was detected and quantified in terms of activity together with initial E2 hormone, unretained compounds, and the total integrated area. This study may provide insight into the development of new and existing water treatment technologies working with radiochemicals in the laboratory environment. The comparison of i) real water samples analysed via LC-MS/MS and ii) radiolabelled samples analysed with the UHPLC-FSA method would be a valuable future contribution to unveiling the complex process of micropollutant removal.

## Supplementary information

Supplementary Information.

## Data Availability

The raw and processed data are available from the corresponding author on request
