# Supplementary material for: Separation and degradation detection of nanogram-per-litre concentrations of radiolabelled steroid hormones using combined liquid chromatography and flow scintillation analysis
Source: Sci Rep. 2020 Apr 27;10:7095. doi: 10.1038/s41598-020-63697-y (PMC7184612; doi:10.1038/s41598-020-63697-y)
Supplement: Supplementary file 1 — Supplementary Information. [file 41598_2020_63697_MOESM1_ESM.docx]

**Electronic Supplementary Information**

**Separation and degradation detection of nanogram-per-litre-concentrations of radiolabelled steroid hormones using combined liquid chromatography and flow scintillation analysis**

**Roman Lyubimenko^1,2^, Bryce S. Richards^1,3^, Andrey Turshatov^1^ & Andrea I. Schäfer^2*^**

^1^ Institute of Microstructure Technology (IMT), Karlsruhe Institute of Technology (KIT), Hermann-von-Helmholtz-Platz 1, 76344 Eggenstein-Leopoldshafen, Germany

^2^ Institute for Advanced Membrane Technology (IAMT), Karlsruhe Institute of Technology (KIT), Hermann-von-Helmholtz-Platz 1, 76344 Eggenstein-Leopoldshafen, Germany

^3^ Light Technology Institute (LTI), Karlsruhe Institute of Technology (KIT), Engesserstrasse 13, 76131 Karlsruhe, Germany

* Corresponding author: Email: Andrea.Iris.Schaefer@kit.edu; Tel: +49 721 608 26906

# Radiochemicals used in the current study

As a demonstration of the structure of analytes, type of labelling, and their properties, the information of steroid hormones was summarised in Table S1.

| **Name (Abbreviation)** | **Chemical structure** | **Molecular weight (g/mol)** | **Solubility in methanol (g/L)** ^1-5^ | **pKa**^6-8^ |
| --- | --- | --- | --- | --- |
| Estrone  (E1) | 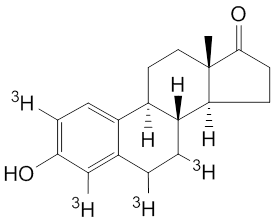 | 270.4 | 5.0 – 5.4  (30°C) | 10.3-10.8 |
| 17ß-estradiol (E2) | 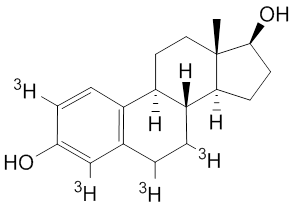 | 272.4 | 35.3 (30°C) | 10.2-10.7 |
| Testosterone (T) | 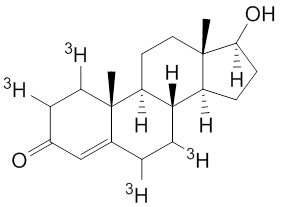 | 314.5 | 224  (30°C) | – |
| Progesterone (P) | 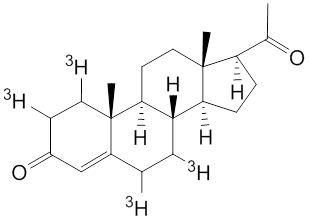 | 288.4 | 54.4 (21°C)  (in ethanol) | – |

**Supplementary** **Table S1.** Chemical structures and properties of tested steroid hormones.

# Error estimation

The total error of count rate ($\Delta CPM$) comprised the uncertainties from the following experimental steps (which are then discussed in more detail in the following paragraphs):

- Sample preparation ($\Delta Prep$ = 5%)
- Experimental system ($\Delta S$ = 1, 8 and 9%) for adsorption, filtration and photocatalysis studies, respectively
- Ultra-high-pressure liquid chromatography (UHPLC) system ($\Delta UHPLC$ = 1%)
- Flow scintillation analysis (FSA) detector ($\Delta Det$ = 12-16 %)

The $\Delta Prep$ term included the variation of pipetting volume (4%) and the very slight decomposition of radiochemicals over a three month period (3%). $\Delta S$ was estimated under assumption of the linear response of hormone removal with uncertainties of system parameters. For static adsorption studies, the $\Delta S$ term (1%) encompassed the variation of such experimental parameters as incubation temperature (1%) and the mass of weighed adsorbent (0.1%). $\Delta S$ of filtration experiments (8%) was reported elsewhere^9^. The $\Delta S$ for photocatalytic degradation experiments evaluated the feed temperature (1%), flow rate (2%), photocatalyst loading (9%), and light intensity (0.1%). The uncertainty of UHPLC system demonstrated a marginal variation of mass of injected analyte (1%) and column temperature (0.4%).

The uncertainty of FSA-detector ($\Delta Det$) and its constituents were calculated via Eqs. (S1) – (S4):

$\Delta Det=\sqrt{{\Delta NC}^{2}+\Delta f_{tot}^{2}}$ , (S1)

$\Delta NC=100\frac{2\sigma_{NC}}{net counts}$ , (S2)

$\sigma_{NC}\mathbf{=}\sqrt{\sigma_{S}^{2}+\sigma_{B}^{2}}$ . (S3)

$\Delta f_{tot}=\sqrt{{\Delta LS}^{2}+{\Delta UHPLC}^{2}}$ , (S4)

where $\Delta NC$ is the uncertainty of net counts obtained from the detector (%), while $\Delta f_{tot}$ (0.6%) is the uncertainty of total flow rate (sum of flow rates of LS and UHPLC pump) through the flow cell of FSA-detector, *net counts* = *counts* (sample) – *counts* (background), $\sigma_{NC}$ is the standard deviation of net counts (2σ corresponds to 95.5% confidence limit)^10^, $\sigma_{S}$ is the standard deviation of the sample counting ($\sigma_{S}\mathbf{=}\sqrt{total counts}$**)** and$\sigma_{B}$ is the standard deviation of the background counting (calculated to be 6 counts/min from blank injections).

$\Delta Det$ depends greatly on the net counts of radiochemicals. It was observed in photodegradation studies where the feed solution was prepared from the E2 batch with different specific activity resulting in different $\Delta Det$ values. The calculated results of total errors ($\Delta CPM$) for E2, unretained compounds (UCs), and metabolite at low and high concentrations (activity) are summarized in Table S2.

| Analyte concentration/ activity | | Net counts (counts) | $\sigma_{NC}$ (counts) | $\Delta NC$ (%) | $\Delta Det$(%) | $\Delta CPM$ (%) | | |
| --- | --- | --- | --- | --- | --- | --- | --- | --- |
|  |  |  |  |  |  | Adsorp-tion | Nanofilt-ration studies | Photo-degrada-tion |
| E2 (ng/L) | 100  100 | 179  287 | 14  17 | 16  12 | 16  12 | 17  – | 21  – | –  13 |
| UCs (Bq/L) | 1·10^3^  1·10^4^ | 11  52 | 4  8 | 51  30 | 51  30 | –  – | –  – | 53  30 |
| Unknown photo-degradation metabolite | | 109 | 11 | 20 | 20 | – | – | 22 |
| Total radio-chromatography run | | 282* | 21* | 15 | 15 | – | – | 18 |

**Supplementary** **Table S2.** The estimation of total error of the count rate of analytes in different example studies.

*The standard deviation of background (y_B_= 35 counts/min, $\sigma_{B}$= 6 counts/min) was evaluated on the basis of 16 min chromatography run (n = 7 blank samples).

# The influence of flow rate of scintillation liquid on the E2 radiochromatograms

To achieve the highest chromatographic resolution and sensitivity (that generally exist as trade-off parameters), the influence of residence time of analyte in the radiodetector (varied via the flow rate of scintillation liquid) was assessed (Fig. S1). Although the retention time of the analyte is not dependent on the LS flow rate, the peak shape estimated as the full width at half maximum (FWHM) was different. The lowest FWHM, as well as the height of the E2 peak (related to method sensitivity), was obtained starting from the flow rate of 4 mL/min. At the flow rate of 3 mL/min, the peak area reached a steady value of 120 counts. As the minimum required HPLC:LS flow rate ratio of 3^11^ was reached, the LS flow rate of 4 mL/min was chosen based on the calculated FWHM and peak area.

**Supplementary** **Figure S1.** Influence of the flow rate of scintillation liquid on the UHPLC-FSA radiochromatogram of E2 (100 ng/L). UHPLC flow rate - 0.4 mL/min, volume of injection - 50 μL, temperature - 30^°^C, isocratic elution of acetonitrile:water (50%:50%).

# The operation parameters optimised during the UHPLC-FSA method development

The matrix of parameter investigated during the method development was demonstrated in Table S3. Liquid scintillation (LS) pump flow rate was fixed from the preliminary tests of E2 injections with isocratic acetonitrile-water (50%:50%) elution (see Fig. S1). The change of gradient elution time was achieved for the methanol-water mobile phase.

| **Parameters to be optimized** | **LS flow rate**  **(mL/min)** | **UHPLC flow rate**  **(mL/min)** | **Gradient time  (min)** | **Column temperature (^°^C)** | **Injection volume (μL)** | **Hormone concentration (ng/L)** |
| --- | --- | --- | --- | --- | --- | --- |
| 1. UHPLC flow rate | 4 | 0.2, 0.25,  0.3, 0.4 | 25 | 50 | 50 | 100 |
| 2. Gradient time |  | 0.25 | 5, 10, 15, 20, 25, 30 | 50 | 50 | 100 |
| 3.Temperature |  | 0.25 | 25 | 30, 40,  50, 60 | 50 | 100 |
| 4. Injection volume |  | 0.25 | 25 | 50 | 10, 20, 50, 100 | 100 |
| 5. Hormone concentration |  | 0.25 | 25 | 50 | 100 | 1 – 1000 |
| **Standard conditions** | 4 | 0.25 | 25 | 50 | 100 | 100 |

**Supplementary Table S3**. The matrix of varied parameters during the development of UHPLC-FSA method

# **Use of** diluted tritium water **in UHPLC-FSA analysis**

The analysis of dead-volume and dead time was achieved via using unretained compound (diluted tritium water, HTO). The elution of HTO at different activities in radiochromatograms was demonstrated in Fig. S2.

**Supplementary** **Figure S2**. UHPLC-FSA radiochromatograms of HTO standards at different volume activities. The elution conditions are given in Supplementary Table S3 as those of standard conditions.

# UHPLC flow rate influence on the radiochromatograms

The influence of the UHPLC flow rate was tested for each hormone and demonstrated on the radiochromatograms plotted in Fig. S3. To closer estimate the separation quality, the radiochromatograms were then processed to calculate the resolution of pairs of close-eluting hormones (E1-E2, E2-T, T-P). The retention time increases with a decrease in flow rate, while the shape of peaks observed as a peak width improves at higher flow rates. The peak area for all hormones remained the same. HTO peaks may correspond to the peaks of unretained compounds observed in radiochromatograms of injected hormones (*t_r_* = 2- 4 min, Fig. S3-S5). However, it is not clear if it is an indication of oxidation (aging) of hormone solution. Here, diluted tritium water can serve as a sign of the impurity of radiolabelled compounds.

**Supplementary** **Figure S3.** Influence of UHPLC flow rate on the elution of ^3^H-labelled E1, E2, T and P. The elution conditions were presented in Supplementary Table S3.

# Influence of methanol-water gradient time on the radiochromatograms

The influence of the gradient time was tested to demonstrate its influence on column selectivity and analyte retention, as illustrated in Fig. S4. The increase of gradient time is expressed via the reduction of slope of the curve for the methanol content in the eluent (Fig. S4a). It is observed that the retention time increases with an increase in time of methanol-water gradient elution (Fig. S4b). Meanwhile, the peak area remains the same, but the FWHM rises from 0.18 to 0.29 min as the gradient time increases

**Supplementary** **Figure S4.** (a) The methanol composition profile at different gradient elution. (b) Influence of gradient time on the elution of ^3^H-labelled E1, E2, T, and P. The elution conditions were presented in Supplementary Table S3.

# Influence of column temperature on the radiochromatograms

The column temperature may affect the retention of analytes. Thus, to demonstrate the changes in peak resolution, the radiochromatograms were first obtained, as plotted below in Fig. S5. With a decrease in column temperature, the analytes are retained more, which is seen from a shift of retention time. The peak area is not dependent on the column temperature. Besides, the column temperature has little effect on the width of peaks.

**Supplementary** **Figure S5.** Influence of column temperature on the elution of ^3^H-labelled E1, E2, T, and P. The elution conditions were presented in Supplementary Table S3.

# Influence of volume of injection on the radiochromatograms

As the peak shape may change at high volume injections (V_inj_>10 μL), the different volumes of injection were varied (Fig.S6). With an increase in the volume of injection, the analyte mass, as well as the peak area, increased. As the retention time is not dependent on the concentration of analytes, the observed shifts were related to the reproducibility error of retention time that lies within 1% (0.1 min of 14 min). The peak width for all hormones increased with larger injection volumes apart from the 100 μL injections.

**Supplementary** **Figure S6.** Influence of volume of injection on the elution of ^3^H-labelled E1, E2, T, and P. The elution conditions were presented in Supplementary Table S3.

# Radiochromatograms of hormone standards for calibration curves

The calibration curves were obtained from a series of standard solutions with known concentrations of hormones. Their radiochromatograms are presented in Fig.S7. The peak areas were calculated for each hormone concentration and plotted against the different concentrations to obtain calibration curves of specific hormones.

**Supplementary** **Figure S7.** The UHPLC-FSA radiochromatograms of ^3^H-labelled E1, E2, T, P at different concentrations used then to reconstruct the calibration curves for each hormone. The elution conditions were presented in Supplementary Table S3.

# Additional information on the estimation of LOD/LOQ

1. The count rate underneath the area of background noise (n = 12 injections) was integrated in ProFSA software.
2. The mean value (counts/min) and standard deviation (counts/min) were extracted from the resulted values of background.
3. y_B_ (mean value of integrated areas of blank injections) and *σ_B_* (standard deviation of integrated areas of blank injections) were used to calculate the *y_LOD_*, *y_LOQ_* (instrument response at concentrations corresponding to LOD, LOQ) using the Eqs. (7) and (8) described in the experimental part.
4. The calibration curves were fitted in linear coordinates and presented in logarithmic coordinates for the sake of clarity. The peak area (counts/min) of the lowest concentration (typically 5 ng/L) used for the fitting procedure (*y_cal.curve_*) was above that of calculated *y_LOQ_* (*y_cal.curve_* > *y_LOQ_*).
5. The upper range concentration was limited by 100 ng/L to avoid the error caused by the overload of column at high concentrations.
6. The obtained fit equations were used to calculate LOD and LOQ.

# References

1. Ruchelman, M. W. Solubility studies of estrone in organic solvents using gas-liquid chromatography. *Anal. Biochem.* **19**, 98-108 (1967).

2. Doisy, E., Huffman, M. N., Thayer, S. A. & Doisy, E. A. Solubilities of some estrogens. *J. Biol. Chem.* **138**, 283-285 (1941).

3. Ruchelman, M. W. & Haines, P. Solubility studies of estradiol in organic solvents using gas-liquid chromatography. *J. Chromatogr. Sci.* **5**, 290-296 (1967).

4. Ruchelman, M. W. Solubility studies of testosterone in organic solvents using gas chromatography. *J. Chromatogr. Sci.* **9**, 235-240 (1971).

5. Sieminska, L., Ferguson, M., Zerda, T. W. & Couch, E. Diffusion of steroids in porous sol-gel glass: application in slow drug delivery. *J. Sol-Gel Sci. Technol.* **8**, 1105-1109 (1997).

6. Perrin, D. D., Dempsey, B. & Serjeant, E. P. *pKa prediction for organic acids and bases*. Vol. 1 (Springer, 1981).

7. Lewis, K. & Archer, R. pKa values of estrone, 17β-estradiol, and 2-methoxyestrone. *Steroids* **34**, 485-499 (1979).

8. Bhandari, A. *et al.* *Contaminants of emerging environmental concern*. (American Society of Civil Engineers, 2009).

9. Imbrogno, A., & Schäfer, A. I. Comparative study of nanofiltration membrane characterization devices of different dimension and configuration (cross flow and dead end). *J. Membr. Sci.* **585**, 67-80 (2019).

10. Malonda, A. G. & Carles, A. G. Radioactivity counting statistics in *Handbook of Radioactivity Analysis* (ed. L'Annunziata, M. F.) Ch. 2, 163-189 (Academic Press, 2012).

11. L'Annunziata, M. F. Flow-cell analysis in *Handbook of radioactivity analysis* (ed. L'Annunziata, M. F.) Ch. 17, 1117-1178 (Academic Press, 2012).
